# A novel use of arterial spin labelling MRI to demonstrate focal hypoperfusion in individuals with posterior cortical atrophy: a multimodal imaging study

**DOI:** 10.1136/jnnp-2015-312782

**Published:** 2016-01-05

**Authors:** Manja Lehmann, Andrew Melbourne, John C Dickson, Rebekah M Ahmed, Marc Modat, M Jorge Cardoso, David L Thomas, Enrico De Vita, Sebastian J Crutch, Jason D Warren, Colin J Mahoney, Jamshed Bomanji, Brian F Hutton, Nick C Fox, Xavier Golay, Sebastien Ourselin, Jonathan M Schott

**Affiliations:** 1 Dementia Research Centre, UCL Institute of Neurology, London, UK; 2 Translational Imaging Group, Centre for Medical Image Computing, University College London, London, UK; 3 Institute of Nuclear Medicine, University College London Hospitals, London, UK; 4 Lysholm Department of Neuroradiology, National Hospital for Neurology and Neurosurgery, London, UK; 5 Neuroradiological Academic Unit, Brain Repair & Rehabilitation, UCL Institute of Neurology, London, UK

**Keywords:** ALZHEIMER'S DISEASE, CEREBRAL BLOOD FLOW, AMYLOID, MRI, PET, LIGAND STUDIES

## Introduction

Posterior cortical atrophy (PCA) is a rare neurodegenerative syndrome, typically due to Alzheimer pathology, characterised by impairments in higher-order visual function and other parieto-occipital skills.^1^ MRI measures of atrophy and ^18^F-labelled fluorodeoxyglucose (FDG) positron emission tomography (PET) measures of glucose metabolism typically show posterior cortical deficits broadly mirroring the focal cognitive deficits.^1^ By contrast, amyloid PET studies demonstrate that fibrillar amyloid is widely deposited across the cortex.^2^ Arterial spin labelling (ASL) is an MRI methodology that uses endogenous arterial blood water as a tracer to quantify cerebral blood flow (CBF).^3^ We aimed to assess the ability of ASL to detect patterns of reduced CBF in PCA, and to compare these results with those from other imaging modalities.

## Methods

Five patients fulfilling clinical diagnostic criteria for PCA,^4^ and five controls attended for three scanning visits usually on consecutive days. On day 1, MRI scans were acquired on a 3 T Siemens TIM Trio scanner with a 32-channel phased array head-coil. Sequences included a sagittal three-dimensional (3D) MPRAGE T1-weighted volumetric scan (acquisition time 9 min 23 s, TE/TR/TI=2.9/2200/900 ms, dimensions 256×256×208, voxel size 1.1×1.1×1.1 mm), and coronal T2 fluid-attenuated inversion recovery (TE/TR/TI=87/9000/2500 ms, voxel size 0.9375×0.9375×5 mm). Perfusion data were acquired using pulsed ASL (FAIR Q2TIPS) with an 8-segment, background-suppressed 3D GRASE imaging readout^5^ (acquisition time 6 min 40 s, TI1/2=800/2000 ms, voxel size 3.8×3.8×4.0 mm, refocusing pulse flip angle 130°, five repetitions). A set of three saturation recovery images (TR=1,2,5 s) with the same readout module was also acquired to generate tissue M0 and T1 maps for CBF quantification. On day 2, each participant underwent a 10 min PET scan 50 min after an intravenous bolus of 300 MBq ^18^F-florbetapir. On day 3, a 20 min PET scan was acquired 30–35 min postinjection of 185 MBq ^18^F-FDG. PET scans were performed on a GE Discovery ST PET/CT scanner, with CT scans acquired immediately before each scan for attenuation correction.

T1-weighted images were processed using a Gaussian mixture model optimised through expectation maximisation to produce probabilistic segmentations of grey matter, white matter and cerebrospinal fluid. Regional labels were produced simultaneously from the integration of the template parcellations and the segmentation^6^ to define (1) a reference region (whole cerebellum) for the PET normalisation; and (2) frontal, medial temporal, lateral temporal, parietal, posterior cingulate and occipital grey matter lobar regions which were subsequently corrected for total intracranial volume. ASL images were aligned to the T1-weighted image using a symmetric affine registration algorithm to reduce the effects of any residual motion.^7^ CBF maps were generated by fitting a derivative of the general kinetic model to the ASL data.^8^ Florbetapir scans were rated amyloid-positive/negative on grey-scaled images according to standard guidelines; FDG scans were rated for regional hypometabolism. Standard uptake value ratios were calculated for each grey matter lobar region and as a composite, using whole cerebellum as the reference region.

Neuroimaging data from each patient were compared against the controls using a modified t test developed to conduct single participant comparisons, by treating the control sample as sample statistics rather than as population parameters.^9^ To explore the regional relationships between the different techniques, z-scores for each modality and each lobe were calculated relative to controls, and linear regression was used across all brain regions to compare imaging modalities.

## Results

**Figure 1:**
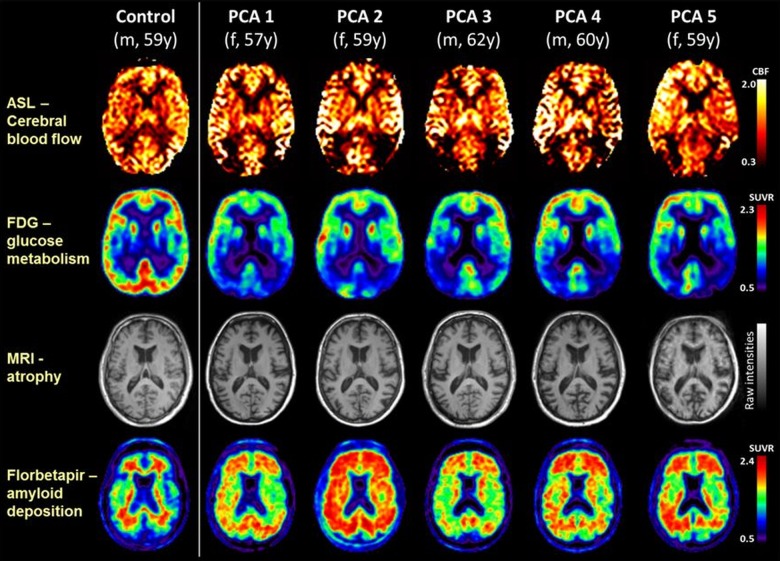
Single-participant axial images for one control participant and five patients with PCA showing cerebral blood flow (ASL), glucose metabolism (FDG-PET), atrophy (structural MRI), and amyloid deposition (florbetapir-PET). For clinical purposes, 18F-florbetapir images should be read on a grey scale. ASL, arterial spin labelling; CBF, cerebral blood flow; FDG-PET,^18^ F-labelled fluorodeoxyglucose positron emission tomography; PCA, posterior cortical atrophy; SUVR, standard uptake value ratio.

Participant demographics and neuroimaging data are shown in online supplementary table S1. At the time of scanning, the patients (3 female, 2 male) had a mean±SD age of 59.4±1.8 years. Single-participant images are shown in the figure 1, alongside the results from a control participant (male, 59 years old) as a reference. All patients were amyloid-positive, with fibrillar amyloid distributed widely across the cortex. Much more focal, posterior cortical reductions in cerebral blood flow, glucose metabolism and atrophy were seen. While atrophy in posterior regions was present in all patients, on visual assessment it was less pronounced than the metabolic deficits assessed using either FDG-PET or ASL.

Quantification of the regional results for each modality is shown in the online supplementary table S1. Compared with controls, there were consistent and significant reductions in grey matter volume and glucose metabolism in the parietal lobe in all patients, and in the parietal, occipital and lateral temporal lobes in the majority. Cerebral blood flow reductions were seen in the parietal and occipital lobes and posterior cingulate for all patients, reaching significance in three. In all cases amyloid deposition was distributed widely across the cortex, including the frontal lobes. Regression analyses showed no significant relationships between amyloid load and any other biomarker. There was no evidence for a relationship between grey matter volume and CBF (p=0.4), borderline evidence for a relationship between grey matter volume and FDG-PET metabolism (p=0.05), but a significant correlation between regional CBF and FDG-PET metabolism (p=0.004, R^2^=0.26).

## Discussion

All individuals in this study had clinical and cognitive phenotypes consistent with PCA, and unequivocally positive florbetapir PET scans consistent with underlying AD pathology. In keeping with previous studies, amyloid deposition was widely distributed across the cortex^2^ while atrophy, hypometabolism and CBF deficits were all more marked in posterior cortical regions.

Previous studies have shown correspondence between the pattern of cerebral hypometabolism measured using FDG-PET and ASL measures of CBF in typical late-onset AD^10^ and semantic dementia.^11^ Here, we extend these findings to PCA, but importantly show that these group level changes extend to individual patients. Posterior cortical CBF reduction was detectable in each of the individual patients with PCA and broadly mirrored by the observed focal patterns of FDG-PET glucose hypometabolism—the gold standard for detecting regional metabolic changes. Both modalities exhibited more extensive changes than expected purely due to the posterior atrophy seen on structural imaging. These data show that ASL, a non-invasive MR sequence which can implemented on standard clinical 3 T MR scanners, can usefully demonstrate CBF changes in individual patients with PCA. ASL may be a useful diagnostic adjunct in suspected PCA—and potentially other focal dementias—where an FDG-PET scan is unavailable.

## Supplementary Material

Web supplement
