# Supplementary material for: A novel use of arterial spin labelling MRI to demonstrate focal hypoperfusion in individuals with posterior cortical atrophy: a multimodal imaging study
Source: J Neurol Neurosurg Psychiatry. 2016 Jan 5;87(9):1032–4. doi: 10.1136/jnnp-2015-312782 (PMC5013120; doi:10.1136/jnnp-2015-312782)
Supplement: Web supplement [file jnnp-2015-312782-s1.pdf]

Supplementary Table: Patient demographics and neuroimaging data

Demographics	**PCA 1**	**PCA 2**	**PCA 3**	**PCA 4**	**PCA 5**	**Controls (N=5) †**
**Age at time of scan, years**	57	59	62	60	59	63.9 (6.4)
**Gender**	F	F	M	M	F	2F, 3M
**Age at onset, years**	55	57	59	53	56	-
Neuroimaging						
**GM volume (% of TIV)**						
Frontal lobe	10.0%	9.3%	9.6%	10.7%	9.1%	10.8 % (0.7%)
Medial temporal lobe	2.0%	2.1%	2.2%	2.0%	1.7% *	2.2% (0.1%)
Lateral temporal lobe	5.1% *	5.0% *	5.5%	4.5% *	3.8% *	5.9% (0.2%)
Parietal lobe	4.7% *	4.8% *	4.3% *	4.4% *	4.6% *	6.3% (0.4%)
Posterior cingulate gyrus	0.4% *	0.4% *	0.4% *	0.5%	0.4% *	0.5% (0.03%)
Occipital lobe	4.4%	4.1%	4.4%	3.7% *	3.0% *	4.8% (0.5%)
**Cerebral blood flow (ml/100g/min)**						
Frontal lobe	35	32	29 *	35	48	39 (4)
Medial temporal lobe	31	30	35	28	38	28 (9)
Lateral temporal lobe	34	39	35	35	43	37 (7)
Parietal lobe	26	21 *	15 *	18 *	31	38 (7)
Posterior cingulate gyrus	35	26 *	30	27 *	36	43 (6)
Occipital lobe	24	28	16 *	16 *	26	41 (10)
Global mean	32	31	28	29	39	37 (8)
**FDG SUVRs**						
Frontal lobe	1.05	1.06	1.06	1.16	1.17	1.22 (0.09)
Medial temporal lobe	0.83 *	0.88	0.85	0.86	0.83 *	0.90 (0.03)
Lateral temporal lobe	0.92 *	1.00	0.93 *	0.88 *	0.88 *	1.12 (0.07)
Parietal lobe	0.84 *	0.91 *	0.79 *	0.80 *	0.84 *	1.22 (0.11)
Posterior cingulate gyrus	0.95 *	0.83 *	1.06 *	1.02 *	0.97 *	1.32 (0.12)
Occipital lobe	0.91 *	1.16	0.89 *	0.85 *	0.73 *	1.19 (0.08)
Global mean	0.94	1.00	0.94	0.96	0.93	1.13 (0.13)
**AV45 SUVR**						
Frontal lobe	1.38	1.68 *	1.41	1.48 *	1.35	1.06 (0.16)
Medial temporal lobe	1.12 *	1.27 *	1.10	1.18 *	1.07	0.95 (0.07)
Lateral temporal lobe	1.41 *	1.68 *	1.36 *	1.41 *	1.35 *	1.02 (0.09)
Parietal lobe	1.41 *	1.63 *	1.35 *	1.41 *	1.31	1.07 (0.12)
Posterior cingulate gyrus	1.53 *	1.65 *	1.48 *	1.45 *	1.23	1.03 (0.10)
Occipital lobe	1.48 *	1.49 *	1.19	1.45 *	1.42	1.09 (0.15)
Global mean	1.38 *	1.57 *	1.33 *	1.40 *	1.30 *	1.03 (0.11)

* significantly different from control values ($p<0.05$); † Mean (SD) of 5 control subjects, TIV – Total Intracranial Volume
